# Neutralization Sensitivity and Evolution of Virus in a Chronic HIV-1 Clade B Infected Patient with Neutralizing Activity against Membrane-Proximal External Region

**DOI:** 10.3390/pathogens12030497

**Published:** 2023-03-22

**Authors:** Wenqi Tang, Zhenzhen Yuan, Zheng Wang, Li Ren, Dan Li, Shuhui Wang, Yanling Hao, Jing Li, Xiuli Shen, Yuhua Ruan, Yiming Shao, Ying Liu

**Affiliations:** 1 State Key Laboratory of Infectious Disease Prevention and Control, National Center for AIDS/STD Control and Prevention, Chinese Center for Disease Control and Prevention, Beijing 102206, China; 2 Changping National Laboratory, Beijing 102200, China

**Keywords:** HIV-1, MPER, envelope, neutralization sensitivity

## Abstract

The membrane-proximal external region (MPER) is a promising HIV-1 vaccine target owing to its linear neutralizing epitopes and highly conserved amino acids. Here, we explored the neutralization sensitivity and investigated the MPER sequences in a chronic HIV-1 infected patient with neutralizing activity against the MPER. Using single-genome amplification (SGA), 50 full-length HIV-1 envelope glycoprotein (*env*) genes were isolated from the patient’s plasma at two time points (2006 and 2009). The neutralization sensitivity of 14 Env-pseudoviruses to autologous plasma and monoclonal antibodies (mAbs) was evaluated. *Env* gene sequencing revealed that the diversity of Env increased over time and four mutation positions (659D, 662K, 671S, and 677N/R) were identified in the MPER. The K677R mutation increased the IC50 values of pseudoviruses approximately twofold for 4E10 and 2F5, and E659D increased the IC50 up to ninefold for 4E10 and fourfold for 2F5. These two mutations also decreased the contact between gp41 and mAbs. Almost all mutant pseudoviruses were resistant to autologous plasma at both the earlier and concurrent time points. Mutations 659D and 677R in the MPER decreased the neutralization sensitivity of Env-pseudoviruses, providing a detailed understanding of MPER evolution which might facilitate advances in the design of HIV-1 vaccines.

## 1. Introduction

The discovery of broadly neutralizing antibodies (bNAbs) has provided new ideas for designing effective prophylactic vaccines because of their ability to neutralize most globally circulating HIV-1 strains and accelerate the elimination of infected cells [1,2]. Despite much effort, bNAbs have only been identified in a small number of HIV-1 infected patients [3]. BNAbs typically develop slowly in vivo during chronic infection, as the viruses diversify under immune pressure and B cell lineages adapt to the evolving viruses. Understanding the valuable characteristics of viruses linked to the generation of bNAbs in HIV-1 infected patients, particularly the key events involved in the coevolution of viral envelope glycoprotein (*env*) and bNAb lineages, may provide important information for the development of promising vaccines that induce full maturation of the bNAb lineages [4,5].

The diversity of HIV-1 Env is related to the maturation and induction of bNAbs, which provide the immune system with abundant antigenic stimulation and increase the potential for the activation of B cell precursors for bNAb generation [6]. Current vaccine candidates lack the ability to elicit the bNAb response, which is likely to be protective against most circulating viral isolates. Deciphering the characteristics of Env evolution under the influence of bNAbs may lead to a better understanding of the processes underlying the interaction of Env and humoral immunity, which would provide novel strategies for HIV-1 vaccine design.

The membrane-proximal external region (MPER) is a conserved motif near the surface of the viral envelope in HIV-1 and plays an essential role in the virus-cell fusion machinery. Several studies have implicated the MPER as a linear neutralizing epitope, which is the target of known HIV-1 bNAbs, such as 4E10, 2F5, CH12, m66, and Z13 [7,8,9,10]. Among these antibodies, 2F5 and 4E10 showed broadly neutralizing activities, with 4E10 neutralizing 98% of the virus isolates with IC50s below 50 μg/mL [11,12]. However, 2F5 and 4E10 have been documented to exhibit cross-reactivity with human autoantigens and trigger a tolerance mechanism to such antibodies throughout the development of MPER-specific B cells [13,14,15,16]. Thus, there have been several attempts to develop vaccines that specifically target the MPER, but only a few of them have generated neutralizing antibodies. However, 10E8 has been reported to be the most efficient neutralizing antibody against the MPER, without cross-reactivity to human autoantigens [9]. Recent research has revealed the structure of the MPER, exhibiting a typical conformational epitope structure upon 10E8 binding, and stable immunogens that expose the MPER through structural simulations have been developed. In combination with these findings, the linear nature of the MPER neutralizing epitope and highly conserved amino acids in the MPER make MPER one of the most promising targets for HIV-1 vaccine development [16,17].

MPER-specific neutralizing responses are represented less compared to other epitopes [18,19], and neutralizing antibodies (NAbs) against the MPER, especially bNAbs, are rarely elicited during natural human infection [4,19]. Therefore, naturally infected HIV-1 patients with NAbs against the MPER can provide more information for the induction and maturation of the bNAbs lineage in the MPER. Thus, exploring the general characteristics underlying the development of viruses in these patients would provide insights for designing immunogens that induce bNAbs.

To investigate the neutralization sensitivity and evolution of the MPER, we focused on a Chinese HIV-1 infected donor, CBJC504, with neutralizing activity against the MPER, which may also provide us an opportunity to study the evolution of the MPER under bNAb pressure. We explored the MPER sequence of viruses in the host using single-genome amplification (SGA) and identified several mutations, including, in particular, two previously uncharacterized mutations, 659D and 677R. We constructed pseudoviruses containing these mutations and tested the neutralization sensitivity to autologous plasma and mAbs. Our results indicate that mutations in 659D and 677R reduce the sensitivity of HIV-1 pseudoviruses to neutralization.

## 2. Materials and Methods

### 2.1. Sample Source and Background

The plasma used in this study was collected from a chronic HIV-1-infected Chinese donor, CBJC504, who became infected with the clade B strain around 1995. He remained therapy naïve before the blood samples were collected, and maintained CD4 T-cell counts above 300 cell/μL at all times. He was identified as a cross-reactive neutralizer, for his plasma showed neutralization activity against HIV-1 BG505 (clade A), JR-CSF (clade B), and HIV-2 C1 (HIV-1 MPER) chimeras. In addition, we isolated NAbs via B cell sorting and screened for their neutralization, and the NAbs also showed neutralization activity against HIV-2 C1 chimeras (unpublished). From 2006 to 2009, the subject had steady CD4 T-cell counts, ranging from 335 to 394 cell/μL, and the viral load slightly increased from 20,700 copies/mL in 2006 to 58,100 copies/mL in 2009 (Table S1). Plasma samples collected from 2005 to 2010, for a total of six time points, were analyzed for neutralization sensitivity, and all *env* genes were derived from the plasma samples collected between 2006 and 2009 by SGA.

### 2.2. Viral RNA Extraction, cDNA Synthesis, and SGA

These processes were performed as previously described [20,21,22]. Briefly, viral RNA was extracted from the plasma using a QIAamp viral RNA mini kit (Qiagen, Valencia, CA, USA) according to the manufacturer’s protocol, and transcribed into cDNA immediately using SuperScript III reverse transcriptase (Invitrogen, Grand Island, NY, USA). The cDNA was diluted in a gradient and 12 replicate wells were set up for each dilution.

The SGA-derived full-length *env* gene was amplified via nested PCR on the diluted cDNA using PrimeSTAR HS DNA Polymerase (Takara, Beijing, China). Most of the positive reactions contained the amplification product of a single cDNA template when the dilution provided less than 30% positive reactions. PCR products from the positive wells were purified using the Qiaquick Gel Extraction kit (Qiagen, Hilden, Germany) following the manufacturer’s instructions.

### 2.3. Functional Plasmid Cloning and Screening

Individual products amplified from cDNA were determined via sequencing on an ABI 3770 Sequencer (Applied Biosciences, Beverly Hills, CA, USA), and the products were cloned into the vector using the pcDNA™ 3.1 Directional TOPO Expression kit (Invitrogen, Waltham, MA, USA). The *env* gene was then inserted with a cytomegalovirus promoter in the proper orientation for protein expression [22]. The cloned product was transformed into *Escherichia coli* JM109 competent cells (Takara, Beijing, China), and screened by culturing the cells on a selective medium containing ampicillin. Positive clones were determined based on the band pattern observed via electrophoresis following double digestion with QuickCut™ Xho I and Hind III (Takara, Beijing, China). If the electrophoresis result after cleavage showed two or more fragments, with one being 5.5 kb and the rest of the bands being at least 3 kb in total, the plasmid was assumed to be successfully ligated.

### 2.4. Pseudovirus Preparation and Titration

The strategy for constructing and generating pseudoviruses has been described previously [6,20,22]. After the *env* gene was cloned into the pcDNA3.1 vector, the Env expression plasmid and the Env-deficient HIV-1 backbone plasmid (pSG3△Env) were cotransfected into 293T/17 cells using the transfection reagent polyethylenimine (Sigma, St. Louis, MO, USA), and the cultures were incubated for 48 h at 37 °C. The obtained pseudovirus was filtered through a 0.45 μm filter, dispensed at 1 mL each, and frozen at −80 °C.

Pseudovirus titers were determined with 50% tissue culture infectious dose (TCID50) using TZM-bl cells, which contain integrated reporter genes for firefly luciferase. Pseudoviruses were horizontally diluted on 96-well culture plates with 11 gradients in quadruplicate wells, and the last column of the 96-well plate was used as a control. TZM-bl cells were then added at 1 × 10^4^ cells/well at a final concentration of 5 µg/mL DEAE-dextran, and the plate was incubated for 48 h at 37 °C with 5% CO_2_. Thereafter, 100 μL of supernatant was removed from each well and 100 µL of Britelite-plus (PerkinElmer, MA, USA) was added. The lysate (150 μL) was transferred to a black 96-well plate for luminescence measurement after 2 min. Wells producing over 10-fold more relative luminescence units (RLUs) than cell controls were rated as positive. Based on the pseudovirus dilution that yields approximately 50,000 to 150,000 RLU equivalents, the TCID values were calculated to estimate the pseudovirus titer.

### 2.5. Neutralization Assays

As previously described, we analyzed the sensitivity of pseudoviruses to plasma and monoclonal antibodies (mAbs) [6,20,22]. Prior to testing, plasmas were heat inactivated at 56 °C for 30 min. Serially diluted (1:3) plasmas or mAbs were incubated at 37 °C for 1 h with 200 TCID50 of the pseudovirus. Fresh TZM-b1 cells were added as previously described, and virus and cell controls were set in the first two columns. After that, the plates were kept in an incubator at 37 °C with 5% CO_2_.

After 48 h of incubation, supernatant (150 μL) was removed and Britelite-plus (PerkinElmer, Waltham, MA, USA) (100 μL) was added and incubated for 2 min. The lysate (150 μL) from each well was transferred to 96-well black solid plates, and luminescence was measured using a Victor 3 luminometer (PerkinElmer, Waltham, MA, USA). The 50% inhibitory dose (ID50) was defined as either the plasma dilution or mAb concentration at which RLU was reduced by 50% compared to that of the virus control wells [23,24].

### 2.6. DNA Sequencing, Alignment, and Analysis

Full-length *env* gene segments were sequenced on an ABI 3770 Sequencer, and amplicons of each sequence were constructed and modified using Sequencher_V5.4.6. The sequences were manually validated in BioEdit_V7.2.6.1 after being manually matched with B.FR.HXB2.K03455, a reference sequence of HIV-1 subtype B. Then all sequences were aligned online using Gene Cutter (http://www.hiv.lanl.gov/content/sequence/GENE_CUTTER/cutter.html (accessed on 16 July 2022)) [25]. DNA sequences were used to construct neighbor-joining evolutionary trees using MEGA 11 with the Bootstrap method and the Kimura 2-parameter model. A bootstrap test was used to determine the dependability of internal nodes, and the sequences at the same time points were marked with the same color. Genetic diversity of the *env* sequence variants was indicated as mean gene distances, which were calculated by MEGA 11.0 using the Bootstrap method and the Kimura 2-parameter model.

### 2.7. Amino Acid Analysis

To compare the consistency of amino acids in the MPER at these two time points, we identified variations in amino acid sites in the MPER. Variant sites were aligned via the Sequence Harmony (SH) method (https://www.ibi.vu.nl/programs/seqharmwww/ (accessed on 23 September 2022)) using SH values to indicate the magnitude of amino acid differences between the two sets of sequences, with higher SH values resulting in smaller differences. We also analyzed the mutations in the MPER to determine the variation in each sequence. Sequence logos were drawn from WebLogo (http://weblogo.berkeley.edu/ (accessed on 26 September 2022)) and information on HIV-1 antibody binding sites and the positions of significant mutations was obtained from HIV Molecular Immunology Database (https://www.hiv.lanl.gov/content/immunology/index.html (accessed on 10 November 2022)) [26].

### 2.8. Simulation of MPER-Antibody Interactions

To analyze the effect of mutations on MPER-antibody binding, we downloaded the crystal structures of antigen-antibody complexes, including 10E8-gp41 (4G6F), 2F5-gp41 (1TJI), and 4E10-gp41 (4XBE) from the RCSB Protein Data Bank (https://www.rcsb.org/ (accessed on 20 November 2022)). We selected the interacting sites around the mutated amino acid within 5 Å and counted the hydrogen bond lengths between them using PyMOL 2.5.

### 2.9. Cells

We used 293T/17 cells obtained from the American Type Culture Collection (ATCC, Manassas, VA, USA) to produce the pseudovirus, and TZM-bl cells from the National Institutes of Health (NIH) to verify whether the pseudovirus was infectious. As a genetically engineered HeLa cell line, TZM-bl cells contained Tat-responsive reporter genes for firefly luciferase and were used to test the neutralizing activity of plasma and mAbs. Both cell lines were maintained in Dulbecco’s modified eagle media with 10% heat-inactivated fetal bovine serum, 100 U/mL penicillin-streptomycin, and 2mM Glutamine at 37 °C with 5% CO_2_.

## 3. Results

### 3.1. Analyses of the SGA-Derived Env Genes

**Figure 1:**
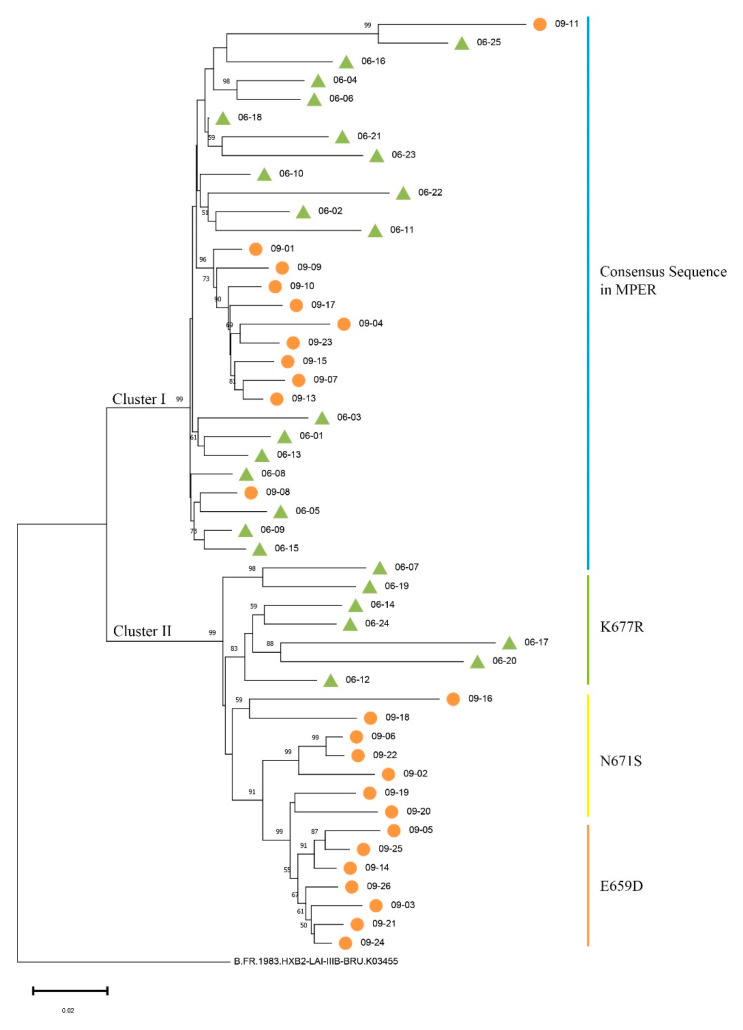
Phylogenetic tree of HIV-1 *env* sequences at two time points in donor CBJC504. The sequences isolated from 2006 plasma were all named starting with 06, and numbered based on the time they were obtained. The sequences isolated from 2009 were named similarly. In the phylogenetic tree, each *env* sequence is represented by a line and sequences from different time points are marked with green triangles (2006) and orange circles (2009). The color of the vertical line in the figure corresponds to the MPER mutation. The blue vertical bar indicates that the sequences are consistent with the consensus sequence in the MPER, and the green, yellow, and orange vertical bars indicate mutations of K677R, N671S, and E659D, in the MPER, respectively.

SGA was used to separate *env* genes from plasma samples collected in April 2006 and June 2009 in order to evaluate Env evolution in donor CBJC504. Fifty full-length *env* genes were amplified and sequenced (Table S2). Phylogenetic analyses were performed, and the results demonstrated that two separate clusters of *env* sequences, Cluster I and II, were formed. The *env* sequences from the two time points were partially intermingled: Cluster I included 18 *env* sequences from 2006 and 11 *env* sequences from 2009, and Cluster II included 7 *env* sequences from 2006 and 14 *env* sequences from 2009 (Figure 1). We also calculated the mean genetic distances of the nucleotide sequences at these two time points, and the distances in 2006 and 2009 were 5.56% ± 0.74% and 8.62% ± 0.66% (mean ± SE) (Table S1), respectively.

**Figure 2:**
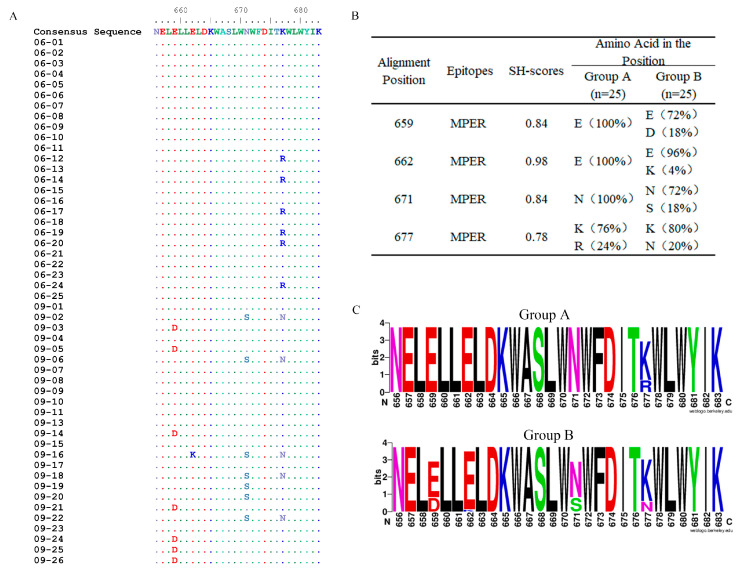
Comparisons of the variation of Env between groups A and B and amino acid sequences with names consistent with nucleic acid sequences. (**A**) Amino acid mutations in the MPER (656–683) at two time points with positions corresponding to HXB2. • (point) represents the same residues with consensus sequence. (**B**) SH values of the mutated positions in the MPER. (**C**) Sequence logos of aa signatures.

Together with the mutations in the MPER, we found that sequences consistent with the consensus sequence in the MPER were in Cluster I, and sequences containing mutations were in Cluster II (Figure 1 and Figure 2A). Furthermore, sequences containing the same MPER mutation subclustered together in secondary evolutionary clusters. For example, in Cluster II, the *env* sequences from 2006 containing the K677R mutation clustered in the same subcluster, and the sequences from 2009 containing the E659D mutations clustered in the same subcluster (Figure 1). It should be noted that 06–07 was the only *env* sequence in Cluster II with the MPER consensus sequence.

### 3.2. Amino Acid Mutations in the MPER

Site-specific mutations in Env are particularly important for immune escape, especially mutations in the MPER (amino acids [aa] 656–683), which contain the epitopes of 2F5 and 4E10/10E8 [9,10,26]. In this study, amino acid mutations in the MPER were analyzed for each sequence, and a consensus sequence in the MPER was produced, which consisted of the most common amino acids at each position in this set of sequences. When compared with the consensus sequence, there was only one mutation in the sequences from 2006 and four in the sequences from 2009 (Figure 2A). This implies that greater diversity and more complexity in the MPER occurred over time in this chronic HIV-1 infected patient.

We then grouped *env* sequences obtained in 2006 and 2009, named group A (*n* = 25) and group B (*n* = 25), respectively, and compared the inconsistent positions in the MPER between these two groups of the sequences using the SH method. An examination of all the positions in the MPER revealed four mutated positions: 659D, 662K, 671S, and 677N/R. The mutation 677R in group A disappeared and was replaced by mutation 677N in group B, and, hence, position 677 had the lowest SH value, implying that it had the greatest variation from 2006 to 2009 (Figure 2B). In group B, mutations 659D and 671S were in the same proportion, but they did not coexist in a single sequence (Figure 2A,B). The highest SH value was at position 662, corresponding to only 1 of the 25 sequences with the 662K mutation. More data are needed to supplement the trend regarding position 662.

We also created sequence logos of mutations in the MPER in these two groups (Figure 2C). Previous studies pointed out that mutations 662K and 671S were linked to an increased resistance to neutralization, and 677N could increase the neutralization sensitivity of viruses [26,27]. We identified two previously uncharacterized mutations in this study, 659D and 677R. Several reports have shown that 659E plays an important role in the interaction between 2F5 and the MPER [28], and 677A increases the sensitivity of 2F5 and 4E10 [29]. Although there have been many studies on these two positions, the mutations 659D and 677R have not previously been documented in natural infection. The effects of mutations 659D and 677R require further verification.

### 3.3. Mutations of 659D and 677R Decreased Neutralization Sensitivity of Pseudovirus to mAbs

To understand whether the mutations affected the neutralization sensitivity of pseudovirus to mAbs, we selected the respective *env* sequences with or without MPER mutations and constructed 26 plasmids of pseudoviruses containing these *env* sequences. After transfection, 14 of the 26 plasmids formed infectious pseudoviruses. Seven of these plasmids did not contain mutations and the other seven contained one of mutations 659D, 671S, 677N, or 677R. However, plasmids with the 662K mutation did not form infectious pseudoviruses, implying that the neutralization sensitivity of this mutant Env cannot be tested.

The neutralization sensitivity of Env pseudoviruses was tested against three mAbs (4E10, 2F5, and 10E8) targeting the MPER. The pseudoviruses without any mutations were sensitive to 4E10, 2F5, and 10E8, with average IC50 values of 0.87 ± 0.89 μg/mL, 1.06 ± 1.25 μg/mL, and 0.11 ± 0.11 μg/mL, respectively (Table 1). This result was consistent with a previous study which found that 10E8 neutralized viruses more effectively and broadly than 2F5 and 4E10 [9].

The mutation 677R increased the IC50 values of pseudoviruses approximately twofold for 4E10 and 2F5, and the pseudoviruses containing the mutation 659D increased the IC50 values up to ninefold for 4E10 and fourfold for 2F5. These results implied that mutations 659D and 677R decreased the neutralization sensitivity of pseudoviruses to mAbs. However, the IC50 values of pseudovirus 09–22, which carries both 671S and 677N mutations, did not change noticeably. Viral mutations are associated with selection from antibodies developed in the host and only a few reduce the neutralization sensitivity of the viruses [27,28,29]. As antibodies targeting the MPER were produced in the host, these observations raised the possibility that the viruses reduce their neutralization sensitivity through the mutations 659D and 677R under pressure from the antibodies.

Differences external to the MPER may affect the exposure and stability of the MPER, further affecting the neutralization sensitivity of the pseudovirus to mAbs [30,31]. The pseudoviruses 06–07, for which sequences were consistent with the consensus sequence in the MPER, were highly neutralization-sensitive to the bNAbs, and pseudovirus 06–14 was sensitive to 4E10, although it contained the 677R mutation. Compared to other pseudoviruses with MPER consensus sequences, 640N and 693I were the unique mutations in 06–07. These mutations may be associated with neutralization sensitivity (Figure S1), as residues 640 and 693 have been reported to have effects on sensitivity [32]. In addition, the sequence of 06–14 contained the largest number of PNGS (potential N-liked glocylation site) in the V1 hypervariable loop among the four pseudoviruses carrying the 677R mutation (Table S3), which was consistent with the previous study that reported that increasing numbers of PNGS in V1 hypervariable loop enhanced virus sensitivity to MPER bNAbs [30].

### 3.4. Sensitivity of Pseudoviruses to Autologous Plasma

To observe virus–antibody interaction and coevolution in the host, we determined the neutralization sensitivity of the 14 pseudoviruses constructed in this study to autologous plasma collected at 6 time points, between 2005 and 2010. The geometrical mean ID50 of the pseudoviruses in 2006 and 2009 for different plasma samples were calculated. Plasma collected from 2007 to 2010 neutralized the pseudoviruses in 2006, and the geometrical mean of ID50 increased from 379 to 614. However, the pseudoviruses in 2009 were neutralized only by plasma from 2010 (Table 2). These results showed that *env* pseudoviruses from earlier time points were effectively neutralized by autologous plasma from later time points, instead of concurrent or earlier plasma, which is consistent with previous studies [20,22,33,34].

In addition, the *env* pseudoviruses without any mutations in the MPER were sensitive to autologous plasma from all time points, but mutant pseudoviruses were resistant to autologous plasma from earlier and concurrent time points, with the exception of pseudoviruses 06–14 and 09–21 (Table 2). Among the four pseudoviruses containing mutation 677R, only 06–14 was sensitive to autologous plasmas, having the highest ID50 values for plasma from almost all time points. Similarly, pseudovirus 09–21, carrying mutation 659D, was also sensitive to autologous plasma. Earlier and concurrent plasma neutralized 09–21, although the IC50 value is relatively low. Since the plasma contained a variety of antibodies against different epitopes, the IC50 value reflected the combined effect of multiple antibodies. The mutation internal or external to the MPER needs further study.

### 3.5. Effects of Mutations E659D and N677R on the Binding of the MPER Epitope to Antibodies in Simulation

**Figure 3:**
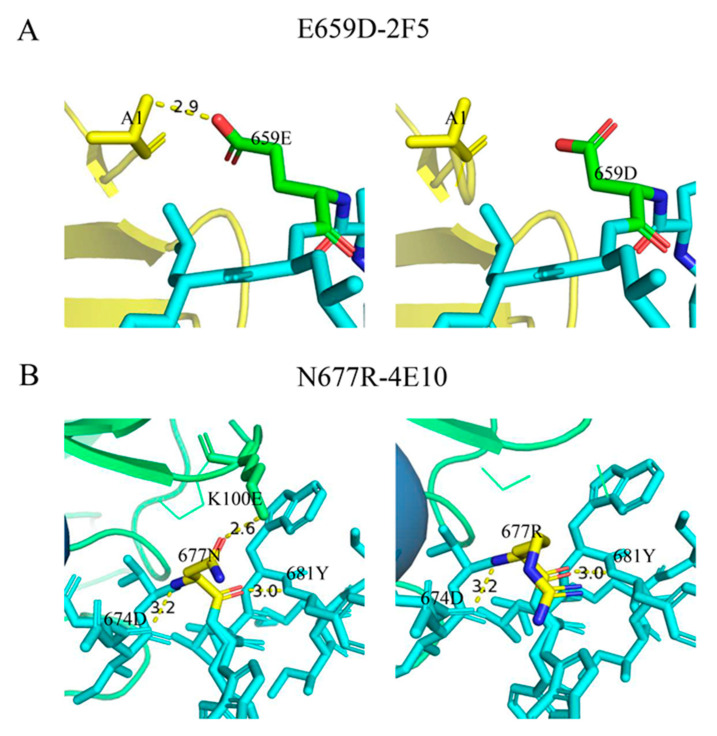
Comparison of interactions between mutated epitopes in the MPER and the antibodies. (**A**) Antibody 2F5 is marked in yellow and the MPER epitopes are marked in blue, except for position 659, which is marked in green. (**B**) Antibody 4E10 and position 677 are marked in green and yellow, respectively.

To investigate the effect of mutations 659D and 677R on the neutralization sensitivity, we simulated the interaction of these two mutated epitopes with the mAbs 2F5 and 4E10. The Fab-gp41 peptide complexes carried either gp41 peptides 656–674 or 671–685, and the interactions of gp41 659D with 2F5, and 677R with 4E10, were simulated [26,32]. We found that 659E contacted A1 on 2F5 with a hydrogen bond length of 2.9 Å; however, once 659E mutated to 659D, there was no contact between these two amino acids (Figure 3A). Compared to glutamic acid (E), aspartic acid (D) is a negatively charged amino acid, which may contribute to a decrease in the contact between residues 659 of gp41 and 2F5. In addition, 677N was in contact with Lys100E^(H)^ (K100E) on 4E10 with a hydrogen bond length of 2.6 Å; however, residue 677R no longer had contact with 4E10 (Figure 3B). Although asparagine (N) and arginine (R) are both polar amino acids, N is uncharged and R is positively charged. As residue 100 on 4E10 is lysine (K), a positively charged amino acid, 677R may interact with Lys100E^(H)^ in a mutually exclusive manner, affecting the binding of residue 677 to 4E10.

The results showed that the mutations of 659D and 677R decreased the contact between gp41 and mAbs, and further decreased the neutralization sensitivity of pseudoviruses to 2F5 and 4E10. We inferred that the viruses in vivo may escape neutralizing stress by mutating to prevent residues in the MPER from binding to neutralizing antibodies.

## 4. Discussion

The diversity of HIV-1 Env is associated with the induction and maturation of bNAbs, which provide the immune system with abundant antigenic stimulation and increase the probability of the activation of the B-cell precursors of bNAbs [6]. Deciphering the characteristics of Env evolution under pressure from bNAbs may help to further understand the mechanisms of interaction between Env and humoral immunity. In vivo neutralization escape and naturally occurring polymorphisms in the MPER are relatively rare; thus, the characterization of natural variants is an important source of structural or function-related information for Env [8,9].

In this study, we explored a chronic HIV-1 infected patient, CBJC504, whose plasma had neutralization activity directed toward the MPER. We obtained 50 *env* gene sequences from the plasma samples collected in 2006 and 2009. A phylogenic analysis of full-length *env* revealed that the sequences formed two distinct clusters: the sequences in Cluster I were consistent in the MPER, and the sequences mutated in the MPER were all in Cluster II. We found four mutated positions (659D, 662K, 671S, and 677N/R) in the MPER, among which mutations 659D and 677R have not been previously reported as natural variations in previous studies.

Residue 677 was ranked the fourth among the ten highest mutations in terms of covariation with potency and structure to 10E8, and it was important for 4E10 interactions with the MPER, which were made by forming a hydrogen bond with the Lys100E^(H)^ of the 4E10 light chain, as it is solvent-accessible in the free form [4,26,35,36]. A previous study showed that K677A increased neutralization sensitivity by tenfold or more for H4K3 and 4E10, and that K677N could also increase sensitivity [27]. In this study, mutation 677R decreased the neutralization sensitivity of pseudoviruses to 2F5 and 4E10.

However, few studies on the MPER epitope have been extended to consider the 659 position. Ofek reported that 659E was the starting site of contact between 2F5 and gp41 at the peptide N-terminus, and that the presence of 659E enhanced the affinity of 2F5 sixfold [28]. In addition, a cyclic peptide that extends the 2F5-acting epitope to the bound β-turn conformation of 659E elicits a high titer of peptide-specific immune responses in guinea pigs, suggesting a role for the residue 659 in antibody formation [37,38]. In this study, the mutation 659D decreased the neutralization sensitivity of pseudoviruses, suggesting that this mutation is more conducive to immune escape.

As for HIV-1 env, residue 662 is very important in neutralization sensitivity to antibodies. Mutation A662G increased the sensitivity tenfold or more for PGXZL1, H4K3, and 4E10 [26], whereas residue 662A and 662K were associated with resistance [27]. In terms of covariation with potency and structure to 10E8, the residue 671 was ranked the sixth among the ten highest; furthermore, the amino acid S at the 671 position were linked to resistance [27,39]. Although we did not package infectious pseudoviruses containing 662K and 671S, we inferred that the mutations 662K and 671S found in this study might be associated with neutralization resistance.

Furthermore, we explored the residues 640 and 693 outside of the MPER, which had unique mutations in 06–07. As one of the signature predictions of 10E8, position 693 is located in the gp41 transmembrane, and together with the Gly-xxx-Gly motif, forms the GGLVG structure, which plays an important role in the self-association of the transmembrane domain. A previous study showed that 693V was associated with an increased neutralization sensitivity to 10E8 [30]. As for 2F5, although there is insufficient evidence to conclude that mutation 640S can increase the sensitivity to 2F5, a high number of PNGS in the V1 hypervariable loop are associated with sensitivity [26], which may explain why 06–14 is sensitive to 4E10.

As a highly conserved motif, the MPER mutated at multiple positions in the host, which may be related to the fact that the patient was chronically infected, suggesting that immune pressure on the MPER epitopes persisted. We also found that the *env* gene with or without mutations in the MPER persisted in the host, and that sensitive and resistant virus strains coexist with bNAbs, resulting in a bNAb:virus equilibrium in which the virus persists but does not produce high levels of viremia [40]. Mutations 659D and 677R may explain the possible mechanism by which the virus adapted to immune stress in this patient.

Overall, our findings indicate that viruses escape immune stress by decreasing neutralization sensitivity through mutations 659D and 677R, which may alter their ability to bind to Nab targeting the MPER. These results may provide more information for the natural mutational characteristics of the MPER.

## 5. Conclusions

In this study, we found that two previously uncharacterized mutations, 659D and 677R, in the MPER could decrease the neutralization sensitivity of Env-pseudoviruses to 4E10 and 2F5. This finding provided detailed information for the evolution of the MPER and might lead to advancements in HIV-1 vaccine design.

## Figures and Tables

**Table 1:** Sensitivity of pseudoviruses to mAbs in the MPER.

Pseudoviruses ID	Mutation Positions in MPER	IC50 (μg/mL)		
		4E10	2F5	10E8
06–01	None	0.60	0.88	0.08
06–02		1.12	0.88	0.11
06–07		2.79	3.85	0.36
06–13		0.35	0.47	0.05
09–04		0.44	0.37	0.05
09–07		0.39	0.50	0.05
09–08		0.37	0.50	0.07
Mean ± SD		0.87 ± 0.89	1.06 ± 1.25	0.11 ± 0.11
06–12	677R	3.89	1.62	0.17
06–14		0.25	1.32	0.16
06–20		2.70	1.35	0.13
06–24		1.72	2.53	0.26
Mean ± SD		2.14 ± 1.54	1.70 ± 0.57	0.18 ± 0.06
09–05	659D	7.87	4.76	0.14
09–21		2.11	0.97	0.14
09–22	671S, 677N	0.74	0.84	0.03

Note: 1. Sensitivity of pseudoviruses to mAbs was measured as IC50 (μg/mL). 2.“None” meant the sequence of pseudoviruses in MPER did not contain mutation.

**Table 2:** Sensitivity of the pseudoviruses to autologous plasmas.

Pseudoviruses ID	Mutation Positions in MPER	ID50 of Autologous Plasmas against Pseudoviruses					
		2005.7	2006.4	2007.4	2008.11	2009.6	2010.11
06–01	None	115	205	847	886	707	611
06–02		125	158	1480	1399	424	498
06–07		521	559	696	1455	1335	1116
06–13		137	179	818	871	380	674
06–12	677R	<20	<20	84	889	1131	597
06–14		60	304	598	727	1294	644
06–20		<20	<20	80	880	1082	572
06–24		<20	<20	150	411	400	401
GEOMEAN (*n* = 8)		53	75	379	882	746	614
09–04	None	28	49	39	22	40	1338
09–07		28	49	54	23	37	835
09–08		51	67	79	42	76	898
09–05	659D	<20	<20	<20	24	<20	312
09–21		25	27	43	68	127	265
09–22	671S, 677N	<20	<20	<20	34	<20	223
GEOMEAN (*n* = 6)		22	28	30	33	34	514

Note: 1. Sensitivity of pseudoviruses to plasma was measured as ID50. 2. The data <20 were converted to 10 to calculate the geometric mean. 3. “None” meant the sequence of pseudoviruses in MPER did not contain mutation.

## Data Availability

All fifty *env* sequences of CBJC504 have been uploaded to GenBank and assigned accession no. OQ389758-OQ389807.

## Supplementary Materials

The following supporting information can be downloaded at: https://www.mdpi.com/article/10.3390/pathogens12030497/s1. Table S1: the profile of the study subject, CBJC504; Table S2: GenBank accession numbers for CBJC504′s *env* gene sequences; Table S3: information of pseudoviruses with 677R mutation in the V1 hypervariable loop; Figure S1: 640-693aa of the sequences of pseudoviruses without mutation in MPER, and the positions correspond to HXB2.
