# Supplementary material for: Neutralization Sensitivity and Evolution of Virus in a Chronic HIV-1 Clade B Infected Patient with Neutralizing Activity against Membrane-Proximal External Region
Source: Pathogens. 2023 Mar 22;12(3):497. doi: 10.3390/pathogens12030497 (PMC10052815; doi:10.3390/pathogens12030497)
Supplement: Supplementary file 1 [file pathogens-12-00497-s001.zip › pathogens-2253443-supplementary.pdf]

Table S1: The profile of the study subject CBJC504.

Sample Data	CD4 + T Cells(cell/μL)	Viral LOAD (Copies/mL)	Mean *env* Distance ± SD （%）	No. of *env* Sequences	No. of Pseudoviruses
11 April 2006	335	20,700	5.56±0.74	25	7
2 June 2009	394	58,100	8.62±0.66	25	6

Table S2: GenBank accession numbers for CBJC504 env gene sequences.

No.	Genebank ID	Names of Sequences	Accession Number
1	BankIt2668815	CBJC504-06-01	OQ389758
2	BankIt2668815	CBJC504-06-02	OQ389759
3	BankIt2668815	CBJC504-06-03	OQ389760
4	BankIt2668815	CBJC504-06-04	OQ389761
5	BankIt2668815	CBJC504-06-05	OQ389762
6	BankIt2668815	CBJC504-06-06	OQ389763
7	BankIt2668815	CBJC504-06-07	OQ389764
8	BankIt2668815	CBJC504-06-08	OQ389765
9	BankIt2668815	CBJC504-06-09	OQ389766
10	BankIt2668815	CBJC504-06-10	OQ389767
11	BankIt2668815	CBJC504-06-11	OQ389768
12	BankIt2668815	CBJC504-06-12	OQ389769
13	BankIt2668815	CBJC504-06-13	OQ389770
14	BankIt2668815	CBJC504-06-14	OQ389771
15	BankIt2668815	CBJC504-06-15	OQ389772
16	BankIt2668815	CBJC504-06-16	OQ389773
17	BankIt2668815	CBJC504-06-17	OQ389774
18	BankIt2668815	CBJC504-06-18	OQ389775
19	BankIt2668815	CBJC504-06-19	OQ389776
20	BankIt2668815	CBJC504-06-20	OQ389777
21	BankIt2668815	CBJC504-06-21	OQ389778
22	BankIt2668815	CBJC504-06-22	OQ389779
23	BankIt2668815	CBJC504-06-23	OQ389780
24	BankIt2668815	CBJC504-06-24	OQ389781
25	BankIt2668815	CBJC504-06-25	OQ389782
26	BankIt2669058	CBJC504-09-01	OQ389783
27	BankIt2669058	CBJC504-09-02	OQ389784
28	BankIt2669058	CBJC504-09-03	OQ389785
29	BankIt2669058	CBJC504-09-04	OQ389786
30	BankIt2669058	CBJC504-09-05	OQ389787
31	BankIt2669058	CBJC504-09-06	OQ389788
32	BankIt2669058	CBJC504-09-07	OQ389789
33	BankIt2669058	CBJC504-09-08	OQ389790
34	BankIt2669058	CBJC504-09-09	OQ389791
35	BankIt2669058	CBJC504-09-10	OQ389792
36	BankIt2669058	CBJC504-09-11	OQ389793
37	BankIt2669058	CBJC504-09-13	OQ389794
38	BankIt2669058	CBJC504-09-14	OQ389795
39	BankIt2669058	CBJC504-09-15	OQ389796
40	BankIt2669058	CBJC504-09-16	OQ389797
41	BankIt2669058	CBJC504-09-17	OQ389798
42	BankIt2669058	CBJC504-09-18	OQ389799

43	BankIt2669058	CBJC504-09-19	OQ389800
44	BankIt2669058	CBJC504-09-20	OQ389801
45	BankIt2669058	CBJC504-09-21	OQ389802
46	BankIt2669058	CBJC504-09-22	OQ389803
47	BankIt2669058	CBJC504-09-23	OQ389804
48	BankIt2669058	CBJC504-09-24	OQ389805
49	BankIt2669058	CBJC504-09-25	OQ389806
50	BankIt2669058	CBJC504-09-26	OQ389807

Table S3: Information of pseudoviruses with 677R mutation in the V1 hypervariable loop.

Pseudoviruses ID	Mutation Positions	V1		
		Sequence Length	Number of PNGS	Charge
06-12	677R	28	3	−1
06-14		37	6	−1
06-20		28	4	0
06-24		34	3	−1

*Note: 1. Sequence length means the length of amino acid in the V1 hypervariable loop.

2. PNGS is an abbreviation for potential N-liked glocylation site.

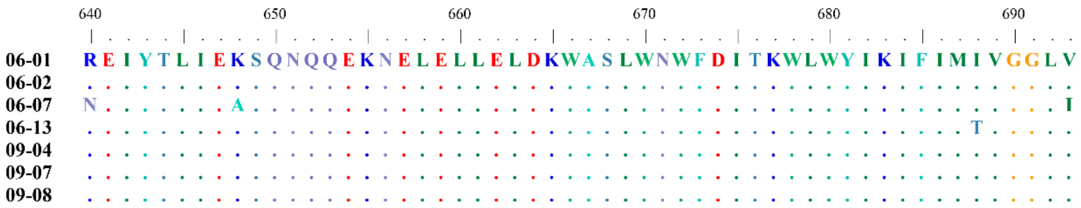

Figure S1: 640-693aa of the sequences of pseudoviruses without mutation in MPER, and the positions correspond to HXB2.
